# Duration of immunity against infectious rhinotracheitis and bovine viral diarrhea after vaccination in calves in southern region of Kazakhstan

**DOI:** 10.3389/fvets.2025.1681624

**Published:** 2025-09-17

**Authors:** Yerbol Bulatov, Zhanna Sametova, Ruslan Abitayev, Asselya Kyrgyzbayeva, Abdurakhman Ussembay, Zhanat Kondibaeva, Zhanat Amanova, Sholpan Turyskeldy, Dariya Toktyrova, Dana Mazbayeva, Kamshat Shorayeva, Kuanysh Jekebekov, Kuandyk Zhugunissov, Kainar Barakbayev, Aslan Kerimbayev, Aralbek Rsaliyev, Yergali Abduraimov, Alina Kurmasheva

**Affiliations:** ^1^Laboratory for Technologies of Cultivation of Microorganisms, Research Institute for Biological, Safety Problems, QazBioPharm, Guardeyskiy, Kazakhstan; ^2^Laboratory for Control of Technologies and Biopreparations, Research Institute for Biological Safety Problems, QazBioPharm, Guardeyskiy, Kazakhstan; ^3^Laboratory for Collection of Microorganisms, Research Institute for Biological Safety Problems, QazBioPharm, Guardeyskiy, Kazakhstan; ^4^Laboratory for Technologies of Finished Forms of Biological Preparations, Research Institute for Biological Safety Problems, QazBioPharm, Guardeyskiy, Kazakhstan; ^5^Laboratory for Monitoring of Infectious Diseases, Research Institute for Biological Safety Problems, QazBioPharm, Guardeyskiy, Kazakhstan; ^6^QazBioPharm, Astana, Kazakhstan; ^7^Department of Biological Safety, Faculty of Veterinary and Zooengineering, Kazakh National Agrarian Research University, Almaty, Kazakhstan

**Keywords:** infectious bovine rhinotracheitis, bovine viral diarrhea, vaccine, prevention, calves, immunity

## Abstract

Some of the most economically significant viral infections affecting cattle globally include infectious bovine rhinotracheitis (IBR) and bovine viral diarrhea (BVD). Both viruses cause a wide range of clinical consequences and significant economic losses. Recent serological surveillance in Kazakhstan showed that these infections persist despite commercial vaccines, underscoring the need for more effective, locally appropriate immunization protocols. This study aimed to evaluate the duration of immunity conferred by a developed associated inactivated emulsion vaccine against IBR (strain “R-93”) and BVD (strain “Oregon C_24_V”), produced by the Research Institute for Biological Safety Problems. In the southern region of Kazakhstan. Of the 12 seronegative for BoHV-1 and BVDV clinically healthy crossbred calves (Friesian-Holstein and Kazakh Whiteheaded) eight were vaccinated, revaccinated, and monitored over a 9-month period for evaluation of the vaccine. Humoral immune responses were assessed using enzyme-linked immunosorbent assay and virus neutralization assays. Specific antibodies to BoHV-1 and BVDV were detected as early as day 7 post-vaccination, with titers peaking at 6.16 log_2_ and 6.24 log_2_, respectively, by day 28, and remaining above protective levels for at least 6 months. At 9 months, a challenge was conducted using virulent strains: no clinical signs or lesions in vaccinated animals, fever and respiratory systems in unvaccinated animals. These findings suggest that the tested inactivated vaccine is safe, immunogenic, and capable of providing sustained protection in crossbred cattle under local climatic conditions. Although the sample size was limited, the results allow us to make preliminary conclusions about the vaccine’s efficacy; however, further large-scale studies are needed.

## 1 Introduction

Infectious bovine rhinotracheitis (IBR) and bovine viral diarrhea (BVD) are among the most prevalent and economically significant viral diseases affecting cattle worldwide (1–8). They are caused by bovine herpesvirus type 1 (BoHV-1) (9) and bovine viral diarrhea virus (BVDV) (10), respectively. Both pathogens are associated with a wide range of clinical manifestations, including respiratory, reproductive, and immunosuppressive disorders in cattle populations (11–13). Modern strategies for the prevention and control of these diseases rely on the use of associated vaccines, designed to induce immunity against multiple pathogens simultaneously (14). Such vaccines offer practical advantages, including a reduced number of injections and decreased animal stress. However, it is important to emphasize that the duration of vaccine-induced immunity can vary considerably depending on factors such as environmental stressors (15), the age of the animal (16) or the vaccine type (17). While modified-live vaccines against IBR and BVD may be associated with adverse effects such as abortion, inactivated vaccines are generally considered safe, including for pregnant cows (18, 19). These factors may directly impact vaccine effecacy under specific regional conditions.

**Figure 1:**
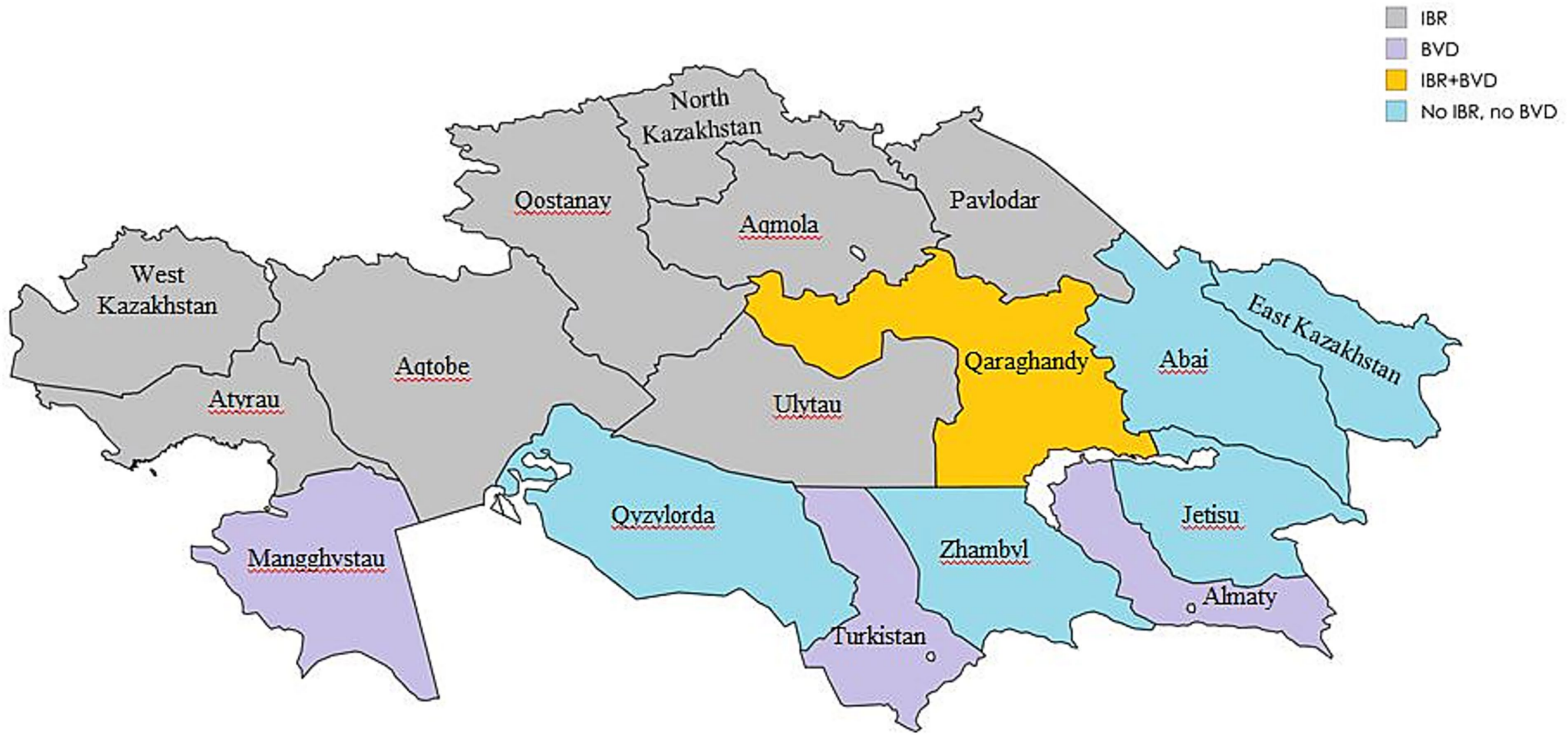
Regional seroprevalence of IBR and BVD in Kazakhstan 2021–2024 (Map generated using mapchart.net).

In Kazakhstan, IBR and BVD control strategies have depended on imported vaccines from Russia and the United States (20). Nevertheless, recent seroepidemiological studies (21, 22) have indicated ongoing circulation of both BoHV-1 and BVDV among cattle herds across various regions of the country (Figure 1).

To solve this issue, the Research Institute for Biological Safety Problems (RIBSP) has developed a domestically produced associated inactivated vaccine against IBR and BVD. The current study aims to evaluate the duration of immunity induced by this vaccine in crossbred cattle (Friesian-Holstein and Kazakh Whiteheaded) raised under typical conditions in the southern region of Kazakhstan. This research provides important insights into the vaccine’s immunogenicity and the longevity of protection in locally adapted cattle breeds.

## 2 Materials and methods

### 2.1 Vaccine and virus strains

The associated inactivated vaccine was developed using the “R-93” strain of BoHV-1 (23) and the “Oregon C_24_V” strain of BVDV (24). Both strains are stored in the Laboratory for Collection of Microorganisms of the Research Institute for Biological Safety Problems (RIBSP). The technology for preparing the vaccine is detailed in the study by Bulatov et al. (25). For challenge studies, the “Oregon C_24_V” strain of BVDV and the “Colorado-1” strain of BoHV-1 (VR-864™), obtained from the American Type Culture Collection (ATCC), Manassas, Virginia, USA (26), were used.

### 2.2 Cell cultures

Two cell cultures were used in this study. The Madin-Darby bovine kidney (MDBK) cell line (CCL-22™) was obtained from ATCC (27). Primary lamb testicle (LT) cell cultures were prepared in the Laboratory for Cell Biotechnology of RIBSP. Parameters and conditions for cell cultivation are described in Bulatov et al. (25).

### 2.3 Animals

Specific consent procedures were not required for this study. There are no client-owned animals. Calves owned by the Department of Experimental Animals of the RIBSP. All animals were pre-screened for antibodies (Abs) against BoHV-1 and BVDV using ELISA. Only clinically healthy and seronegative *Bos taurus* calves were enrolled to exclude interference from pre-existing immunity. 12 clinically healthy and seronegative animals aged 6–12 months were selected. The cohort included 7 heifers and 3 bulls. All animals had ad libitum access to feed and water throughout the experiment. Of the 12 calves enrolled in the study, eight were allocated to the vaccination group. The vaccinated animals were used to assess both the immunogenicity and protective efficacy of the vaccine. While four calves were left unvaccinated and served as controls in the challenge experiment.

### 2.4 Vaccination protocol

8 calves were vaccinated intramuscularly in the middle third of the neck with 2 mL of the vaccine. A booster dose of 2 mL was administered 21 days after the primary vaccination.

### 2.5 Serum sample collection

Blood samples were collected prior to vaccination (day 0), weekly for the first 5 weeks, and then monthly for 9 months to evaluate the duration of the immune response. Blood was drawn from the jugular vein using vacutainer tubes with gel and clot activator. Tubes were labeled with serial numbers, and their decoding (ear tag numbers) was recorded in a special journal. After collection, samples were incubated at 37 °C for 1 h, then cooled to 2–8 °C to allow clotting. Serum was separated by centrifugation at 3000 rpm for 15 min and stored at −20 °C until analysis.

### 2.6 Serological analysis

Abs against BoHV-1 were qualitatively detected using the “PrioCHECH™ BHV-1 gB ELISA Kit” (Thermo Scientific, Massachusetts, USA), and BDBV Abs were detected using the “BVD-80 antibody ELISA kit” (RingBio, Beijing, China). Reactions were performed according to the instructions of the kit manufacturers. Optical density was measured at 450 nm using a Multiskan SkyHigh microplate spectrophotometer (Thermo Scientific, Massachusetts, USA). Ab titers were also measured quantitatively using virus neutralization assays (VNA) based on the neutralization of cytopathic effect, as described by Raizman et al. (28). MDBK cells were used for BoHV-1 VNA, and LT cells for BVDV VNA.

### 2.7 Challenge study design

To assess vaccine efficacy, a challenge study was conducted 9 months post-booster. The timing was based on expected protective titers, as described by Frey (29), where protective thresholds were VNA ≥ 2 log_2_ for BoHV-1 and ≥3 log_2_ for BVDV. In our study, similar titers were observed at 9 months post-vaccination, prompting challenge initiation.

The ear tag numbers of the vaccinated calves were randomly assigned using the GraphPad QuickCalcs randomization tool (30) into 2 groups: Group A (*n* = 2): vaccinated, challenged with strain “Colorado-1” of BoHV-1; Group B (*n* = 2): vaccinated, challenged with strain “Oregon C_24_V” of BVDV. The numbers on the ear tags of 4 non-vaccinated calves were randomly assigned into 3 groups: Group C (*n* = 1): unvaccinated, challenged with strain “Colorado-1” of BoHV-1; Group D (*n* = 1): unvaccinated, challenged with strain “Oregon C_24_V” of BVDV; Group E (*n* = 2): unvaccinated, unchallenged. The challenge inoculum was administered intranasally via animal-specific endotracheal tubing (Raino LLP, Almaty, Kazakhstan) at a dose of 1.5 mL/nostril. Clinical signs (fever, nasal discharge, conjunctivitis, appetite loss) were monitored daily for 10 days post-challenge. Veterinarians conducting the challenge were not blinded to group allocation; however, observers recording clinical signs were blinded to minimize bias. To ensure observer blinding, all calves were identified by serial numbers. Observers were instructed solely to monitor and record clinical signs without access to vaccination records.

On the 5th day after challenge, one calf from Group C was humanely euthanized in accordance with the AVMA Guidelines for the Euthanasia of Animals, 2020 and Large Animal Humane Euthanasia Guidelines, provided by UW School of Veterinary Medicine, 2024, to alleviate suffering. The calf was rendered unconscious by intravenous administration of xylazine (0.1 mg/kg body weight), followed by midazolam (0.1 mg/kg body weight). Once complete loss of consciousness was confirmed (no response to tactile stimulation), exsanguination was performed by transection of the carotid arteries and jugular veins.

### 2.8 Hematological evaluation

Hematological analysis was not included in the initial experimental design. It was performed *ad hoc* after observing severe clinical disease in the unvaccinated calf challenged with BoHV-1. Blood samples were collected on the 5th day after infection from Groups A, C, and E for complete blood count (CBC) analysis using the Abacus Junior5 Vet hematology analyzer (Diatron MI Zrt, Budapest, Hungary) (31). This time point was selected because it coincided with the peak of clinical sings followed by euthanasia. Parameters measured included erythrocyte (RBC), leukocyte (WBC) and platelet (PLT) counts, hemoglobin concentration (HGB), mean corpuscular volume (MCV), mean corpuscular hemoglobin (MCH), mean corpuscular hemoglobin concentration (MCHC).

### 2.9 Ethical approval

This study did not involve any client-owned animals. All animals used were owned by the Research Institute for Biological Safety Problems and were managed solely by institutional staff. All procedures complied with animal welfare regulations and biosafety protocols approved by the RIBSP Ethics Committee (Approval No. 1–14-07-2023), in accordance with national standards for the care and use of experimental animals, AVMA Guidelines for the Euthanasia of Animals, 2020.

### 2.10 Statistical analysis

Data were analyzed using the Mann–Whitney U test, suitable for small sample sizes (*n* < 30) and non-parametric distributions. A *p*-value < 0.05 was statistically significant. GraphPad Prism version 8.0.1 (GraphPad Software, San Diego, USA) was used for all statistical analyses. Both male and female calves were included in the study; however, due to the small number of animals per sex, no stratification by sex was applied during data analysis. No additional statistical analyses were performed; therefore, the conclusions drawn from data should be considered preliminary.

## 3 Results

### 3.1 Serological analysis

**Figure 2:**
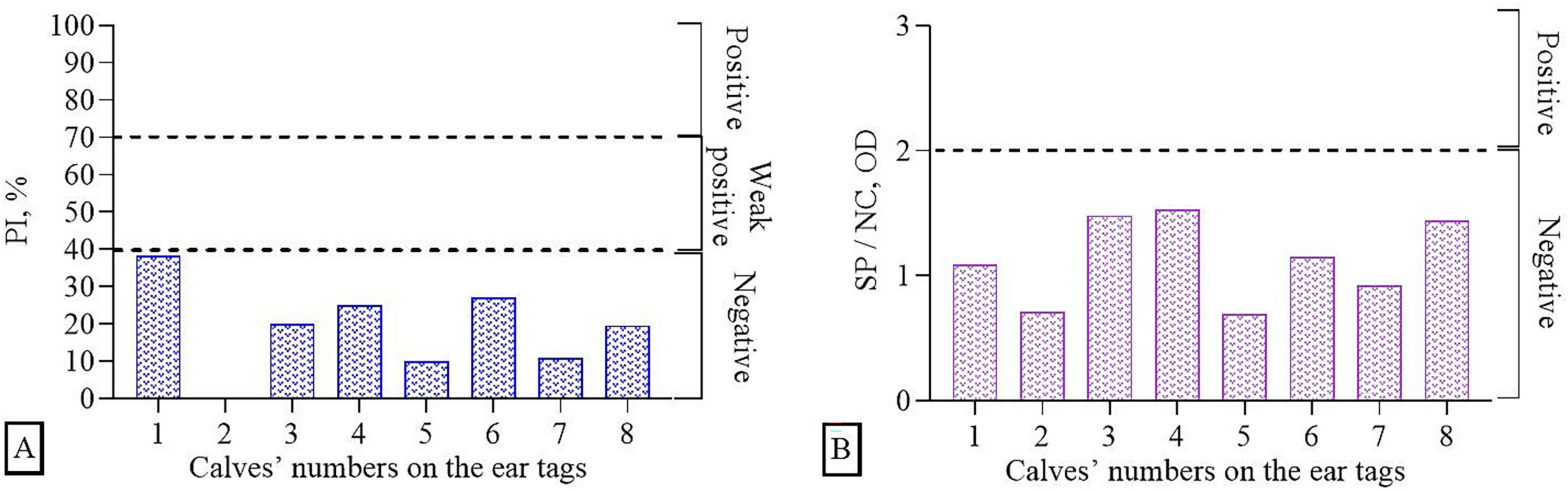
Determination of immune background (presence of Abs in serum samples prior to the experiments) using ELISA: **(A)** BoHV-1, **(B)** BVDV.

Before vaccination, serum samples were collected from 8 calves and tested for the presence of Abs to BoHV-1 and BVDV using commercial ELISA kits (Figure 2). It was found that all samples (*n* = 8) were negative for the IBR virus, i.e., they did not have specific Abs, since the mean of percentage inhibition (PI) values was ≤40%. In the studies of samples for the presence of Abs to the BVD virus, the absence of Abs was also revealed, since the ratio of the optical density of the sample (SP) / the optical density of the negative control (NC) was < 2. Thus, prior to immunization (day 0), no specific Abs against BoHV-1 and BVDV were detected, confirming the absence of pre-existing immunity.

The general health condition of the animals after vaccination remained satisfactory. No local reactions in the form of swelling at the injection sites or no general reactions in the form of fever, conjunctivitis, and serous nasal discharge were observed.

**Figure 3:**
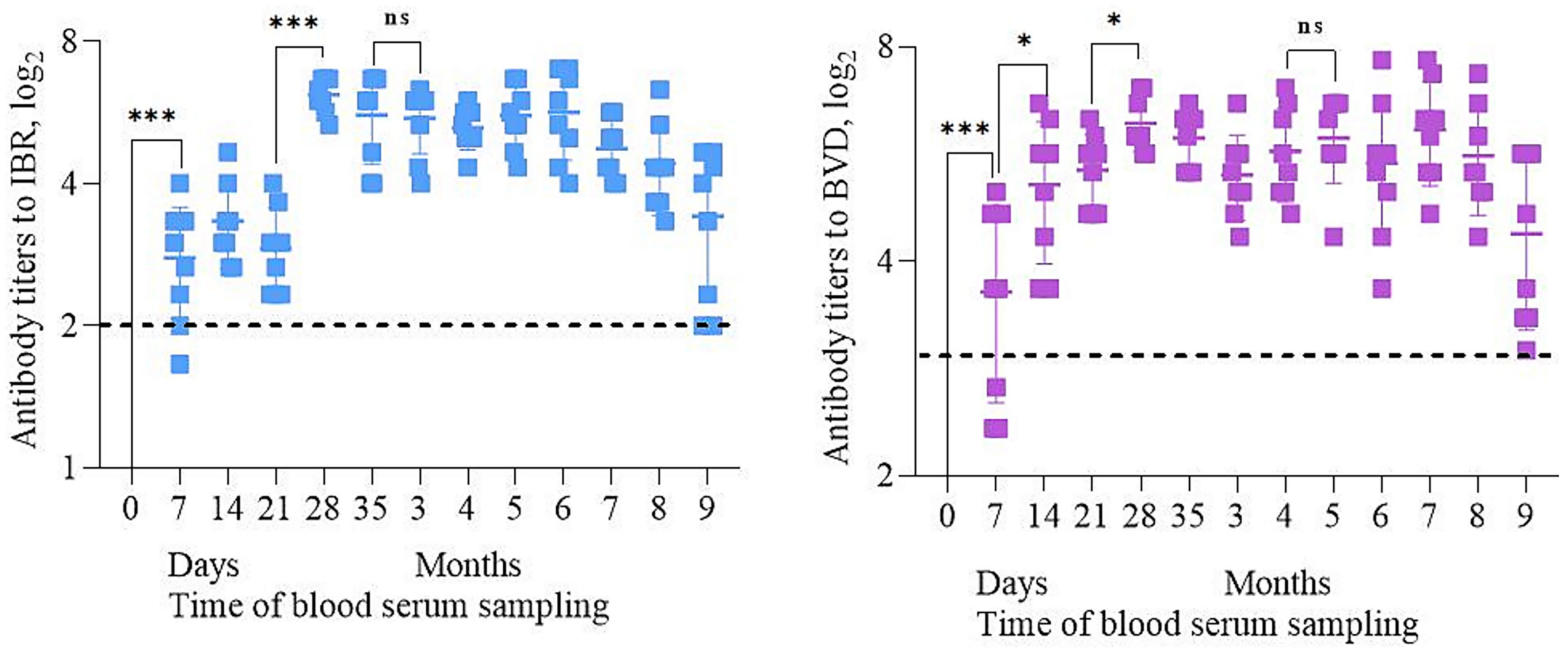
Dynamics of Abs titers to BoHV-1 and BVDV during 9 months post-vaccination period. * – *p* < 0.02; *** – *p* < 0.002; ns – *p* > 0.05. Dotted lines represent minimum protective levels (log_2_) of Abs for each virus (29).

The most significant changes (*p* < 0.05) in Abs titers were noted on days 7 and 28 of the post-vaccination period (Figure 3). By day 7, VNA results showed a measurable increase in Ab titers was observed, indicating the formation of active immunity. The Ab titers were 2.74 log_2_ for BoHV-1 and 3.62 log_2_ for BVDV. A similar trend is observed on the 28th day, i.e., 7 days after revaccination, when the titer of Abs reaching 6.16 log_2_ for BoHV-1 and 6.24 log_2_t for BVDV. Protective Ab levels were maintained throughout the 9-month observation period, consistent with the thresholds reported by Frey (29). By the end of this period, some calves show a decrease in Ab titers, but the levels remained above or near the minimum protective level.

### 3.2 Challenge infection of calves

**Figure 4:**
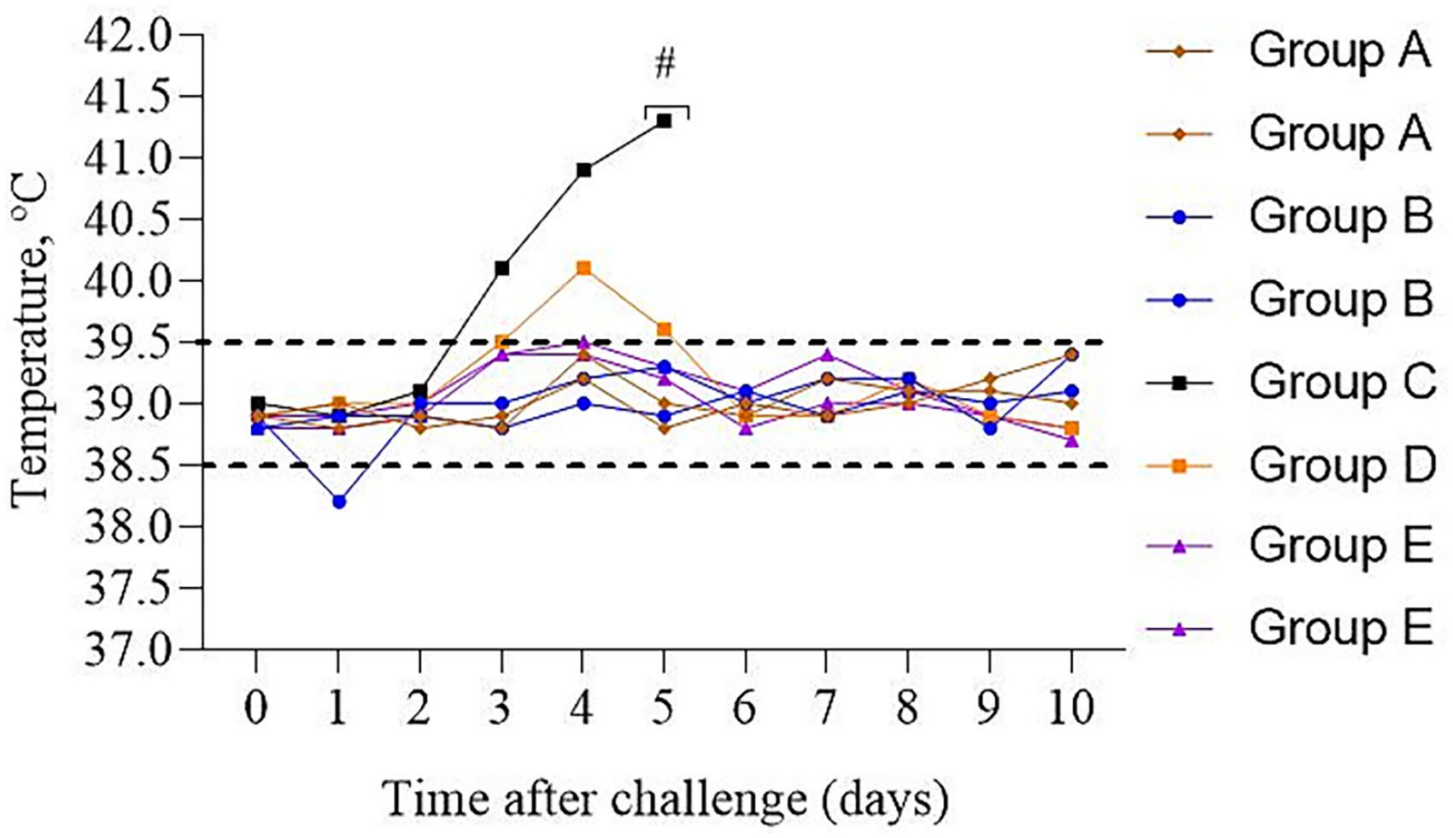
Dynamics of the temperature changes in calves after experimental challenge. Group A – vaccinated, challenged with BoHV-1; Group B – vaccinated, challenged with BVDV; Group C – unvaccinated, challenged with BoHV-1; Group D – unvaccinated, challenged with BVDV; Group E – unvaccinated, unchallenged; # – calf euthanasia. Dotted line – normal physiological temperature range for calves (43).

After a challenge during 10 days of observation, vaccinated cattle from Groups A&B (*n* = 4) remained clinically healthy, the temperature of body was within the physiological norm (Figure 4), no discharge from the mouth, nostrils or eyes was detected. Appetite remained normal.

**Figure 5:**
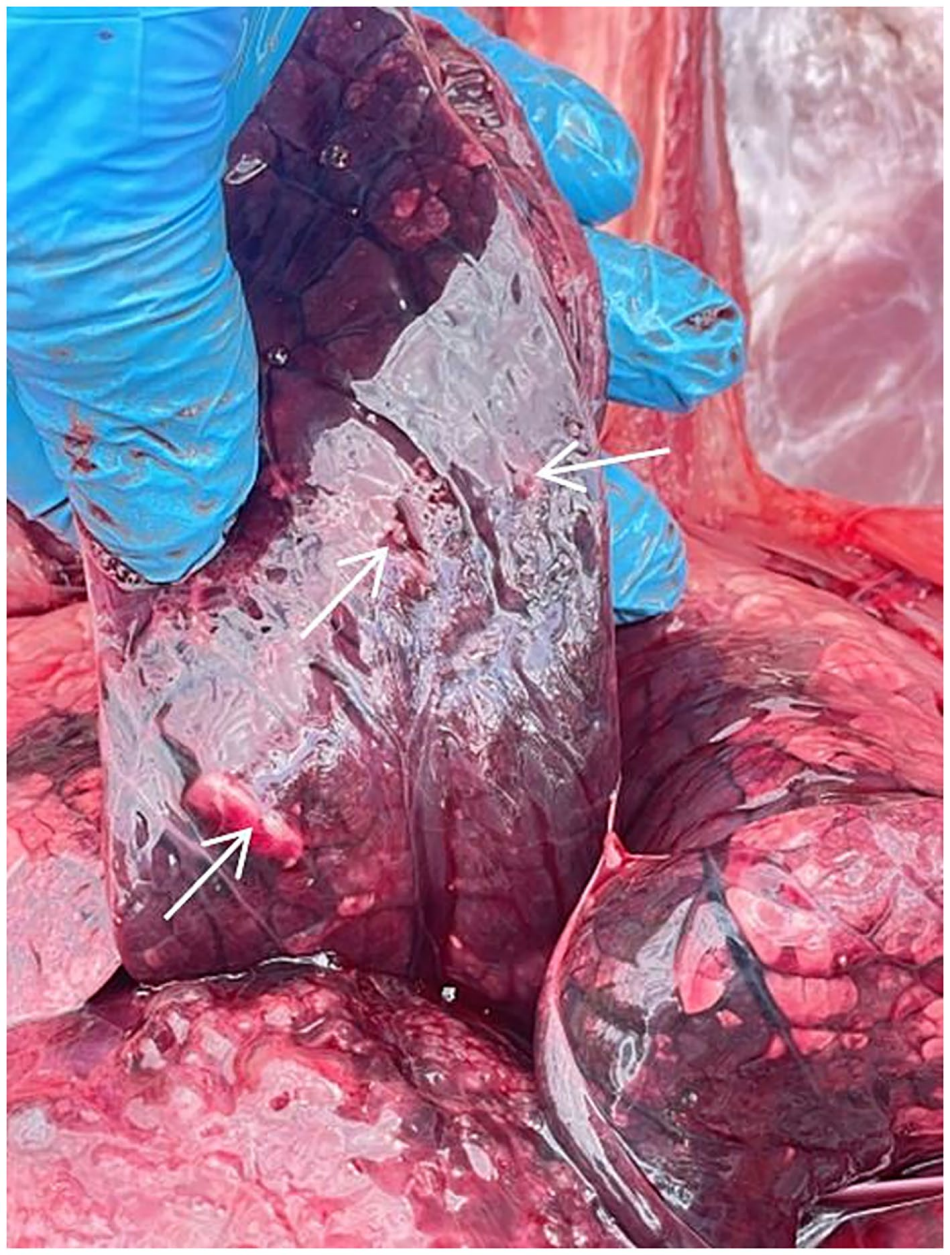
Necropsy of the calf from Group C. Arrows indicate nodules in the lung tissue.

In contrast, the unvaccinated calf from Group C had a cough and sneezing on the 2nd day, and inflammation of the mucous membrane of the eye, loss of appetite, and a body temperature of 40.1 °C were noted on the 3rd day. A fever of 41.3 °C and a severe cough were recorded on the 5th day. Due to the deterioration in the general physiological conditions of the calf, humane euthanasia was performed to alleviate suffering. A post-mortem assessment of the respiratory system was performed (Figure 5). Visible pathological changes were found in the lungs, round, dense nodular formations. The temperature of calf from Group D increased on the 4th day; however, no other clinical signs were found, as in Group C.

**Figure 6:**
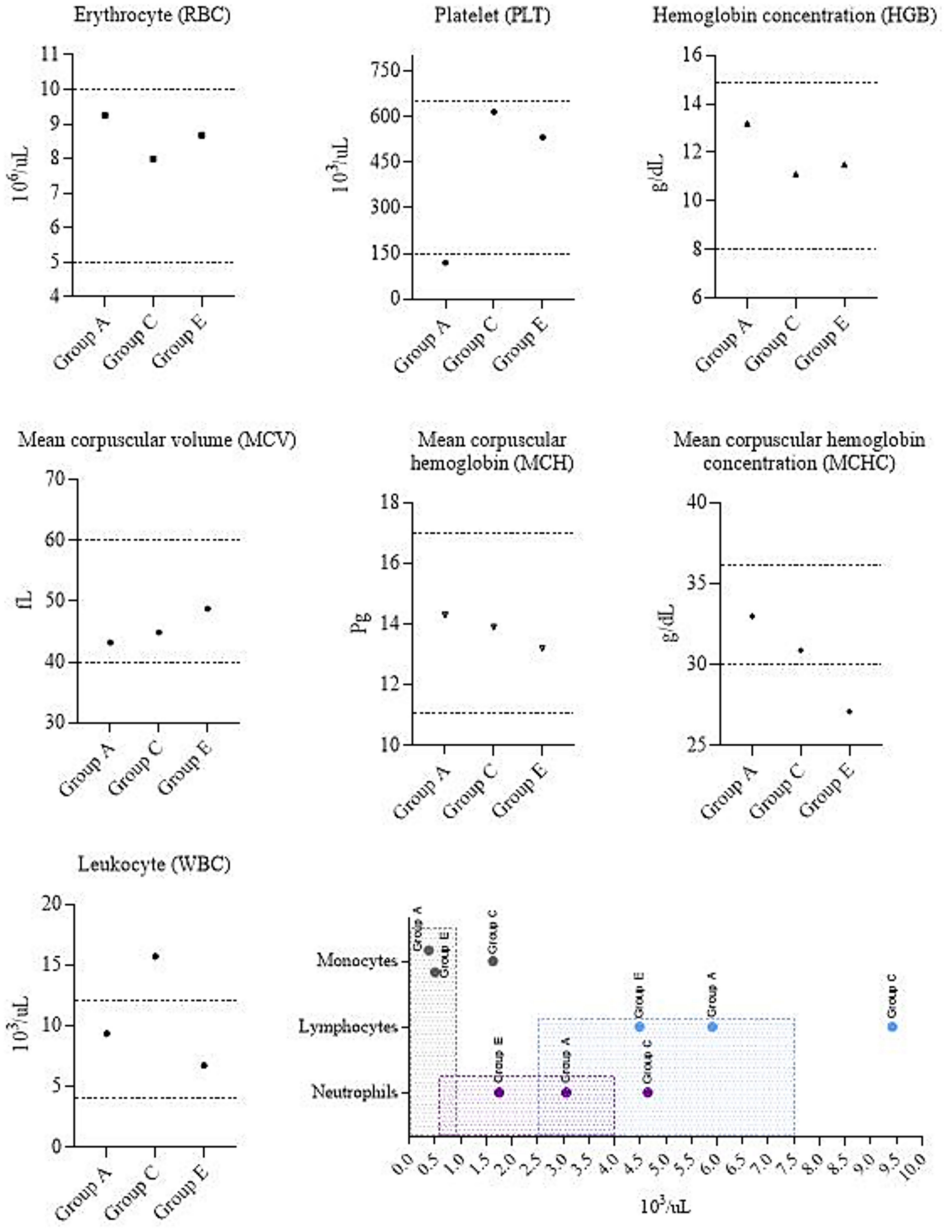
Complete blood count. Group A – vaccinated, challenged with BoHV-1; Group C – unvaccinated, challenged with BoHV-1; Group E – unvaccinated, unchallenged. Dotted lines and shaded areas represent reference values (50).

Due to the euthanasia of the Group C calf on day 5 post-infection, blood samples were collected from one animal each from Group C, A (as it was experimentally infected with BoHV-1), and Group E (control) for the purpose of hematological analysis and comparison with reference values (Figure 6). The vaccinated calf from Group A had a platelet (PLT) count level below the reference interval. A similar trend is observed in mean corpuscular hemoglobin concentration (MCHC) values in the control calf from Group E. In the euthanized calf (Group C), the leukocyte (WBC) values, including neutrophils, lymphocytes and monocytes, were elevated beyond normal limits.

## 4 Discussion

The prevalence of IBR and BVD among livestock is quite high (2–8). All breeds and ages are susceptible to IBR, but it is most often registered among adult livestock (5). Ortiz-González et al. (6) found that seroprevalence was highest in cattle aged >4 years. Clinical signs of BVD are recorded, including in newborn calves (32). Cattle infected with BVDV are highly prone to secondary bacterial infections (33). In our studies, only two viral pathogens – IBR and BVD – were investigated. Thus, evaluating the overall impact of secondary infections, including bacterial ones, was beyond the study’s scope. Moreover, during the experiments calves used were <2 years of age.

According to the statistics committee, as of February 1, 2025, approximately 4.35 million heads of cattle were registered in Kazakhstan (34). The largest number of cattle is registered in the East Kazakhstan, Almaty and Turkistan regions, and the lowest concentration is kept in the Mangghystau and Atyrau regions (20). Despite the use of imported vaccines, seropositive to IBR and BVD animals continue to be detected (21, 22), which indicates the persistent circulation of both viruses. Although no vaccine is considered completely effective (35), the duration of immunity may vary depending on vaccine the type (17). It should be noted that the use of attenuated vaccines for the prevention of viral diseases of cattle, while immunogenic, may poses a significant risk. In contrast, inactivated vaccines are safe and recommended for immunization of cattle, including pregnant cows (36, 37). A study by Peters et al. (38) assessed the duration of immunity provided by the Rispoval 4 vaccine against BoHV-1 and three additional bovine viruses, which was found to be more than 6 months.

We conducted studies to evaluate the duration of immunity after vaccination with the developed associated inactivated vaccine against IBR and BVD under the conditions of the southern region of Kazakhstan. The experiments involved 12 heads of cattle of the local mixed breed (Friesian-Holstein and Kazakh Whiteheaded) in the Zhambyl region from June 2024 to March 2025. Characteristic features of the climate of the Zhambyl region are significant aridity and continentality with minimal precipitation and temperature extremes: summers are very hot, and winters average −8, −12 °C (39, 40). According to Okunev (41), local crossbred cattle and the Kazakh Whiteheaded breeds a less susceptible to BVDV compared to imported purebred animals in North Kazakhstan.

Serology is often used to diagnose infectious diseases (42). ELISA confirmed that the selected calves had no previously acquired immunity to the IBR and BVD. That is, all 12 calves were not vaccinated before the experiments and were seronegative for both viruses. Also, animals did not show clinical signs of infectious diseases. VNA found that a specific immune response began to form as early as 7 days after primary vaccination. Abs titers reached a maximum between days 28 and 35, indicating full activation of humoral immunity. Abs to both viruses remained at a level exceeding the protective titer for at least 6 months. In some animals, Abs were detected up to 9 months.

Based on the results of the control infection of vaccinated and unvaccinated calves, the following can be identified. Normally in mammals, the reference value of temperature is in the range of 36.5–39.5 °C. During infections the set point is increased. According to Sjaastad et al. (43), the normal interval of temperature for a calf is 38.5–39.5 °C. Allan et al. (44) reported that animals affected by IBR developed severe clinical disease characterized by widespread respiratory tract lesions at autopsy. According to Evstifeev et al. (45), the main pathological changes in BVD are localized in the digestive tract; the oral cavity is hyperemic, covered with erosions and ulcers. Prevention of fever is considered a general parameter determining clinical protection of cattle from experimental BVD infection (46).

In challenge studies, no clinical signs of the diseases were detected in vaccinated calves of Groups A&B (n = 4) during a 10-day observation: body temperature stayed within the physiological norm, no changes in behavior; appetite and general condition were satisfactory. Previously, the safety of the vaccine was presented by Kondibaeva et al. (47). In contrast, in the unvaccinated calf from Group C, challenged with the BoHV-1, deterioration of the condition (including fever peaking at 41.3 °C, respiratory distress, and appetite loss) was observed for 3 days (from 3 to 5 days after infection). The incubation period for BoHV-1 typically ranges from 2 to 4 days (48). It is worth noting that we have chosen the following methods of anesthesia and euthanasia: xylazine, midazolam, and exsanguination. This approach was chosen due to the limited availability of ketamine and barbiturates in Kazakhstan. Due to this, we were unable to conduct a direct comparison between protocols. Importantly, based on our observations, no artifacts attributable to the used method were observed that could have affected the post-mortem or hematological outcomes. Necropsy revealed necrotic changes and nodules in the lungs. Similar data were obtained in cattle infected with IBR; however, mycoplasma co-infection has also been detected (49). In an unvaccinated calf from Group D, challenged with the BVDV, an increase of temperature to 40.1 °C was noted on the 4th day; but other clinical symptoms of infection were not detected. Such differences in experimentally infected animals may be due to the fact that BoHV-1 is primarily associated with acute respiratory disease (44), whereas BVDV often causes predominantly gastrointestinal effects (45). These factors likely contributed to a milder clinical outcome in the Group D calf.

The hematological profile reflects the state of health and allows assessing the presence of inflammation. Therefore, we additionally performed a hematological analysis. Results were compared with the established reference values for calves (50). Based on these parameters, Barsukova et al. (51) also identified complete blood count values that fall within the specified range. Erythrocytes (RBC) are responsible for gas exchange, transporting oxygen and carbon dioxide. A complete assessment of RBC includes the mean corpuscular hemoglobin concentration (MCHC). In our study, this parameter was reduced in the calf of Group E. A decrease of MCHC possibly suggesting iron deficiency or anemia (52). Platelets (PLT) play an important role in hemostasis. Low PLT count (as in calf from Group A) can be caused by a number of reasons (52). According to Roland et al. (53), examples of infectious diseases causing thrombocytopenia can be salmonellosis, leptospirosis, and babesiosis. Hence, it cannot be concluded definitively that BoHV-1 alone caused the platelet decrease. Leukocytes (WBC) play an important role in immune protection. Increased WBC in the calf from Group C can indicate stress, excitement, or infectious processes. It is worth excluding bovine leukocyte adhesion deficiency (BLAD), since it is diagnosed when the neutrophil count is more than 40,000 cells/μl, while the changes in the calf of Group C were less than 10,000 cells/μl, which is significantly less for BLAD but exceeds the reference values. Monocytosis can also be caused by necrosis (53). Collectively, clinical signs and pathological changes may diagnosis of IBR disease in this animal.

This study has several limitations:

1. Although we followed ARRIVE 2.0 guidance (54), but changes were made in item 2b, which requires justification of sample size. The power analysis was performed using G*Power 3.1.9.7 (Heinrich Heine Universität Düsseldorf, Düsseldorf, Germany). Test family: F test, ANOVA fixed effects, omnibus with large effect size (*f* = 1.0), *α* = 0.05, power = 0.8, for ANOVA of 5 groups. The required sample size was estimated as *n* = 20. However, due to the fact that the developed vaccine is at the experimental stage, in our research work on vaccination and evaluation of the duration of immunity of cattle, the number of test animals was limited and amounted to 12 heads.
2. In the present study, both male and female calves were included. However, due to the small sample size, animals were not stratified by sex during data analysis. This limitation should be considered when interpreting the vaccination outcomes, as sex-related differences in immune response cannot be ruled out. Given the restricted sample size, no additional inferential statistical tests were feasible, so findings should be interpreted with caution and considered preliminary until validated in large-scale studies.
3. Hematological evaluation was performed only once on day 5 post-infection, and was not part of the original study design. Although this provided useful comparative data on IBR, calves from the B&D groups that were challenged with BVDV were not included in the hematological analysis; that limits conclusions regarding BVD-associated blood parameters. Besides, continuous hematological monitoring would have offered a deeper understanding of disease dynamics. Future studies with larger animal groups will include hematological measurements at multiple time points.
4. Even if calves were suspected of having possible anemia or other infections, no further diagnostic investigations (e.g., viral isolation, PCR, or bacteriological cultures) were performed to determine the causes of the abnormal hematological findings observed in the Group A&E calves. Likewise, although post-mortem examination of the Group C calf revealed round, dense nodular formations in the lung, no specific testing for mycoplasma or other bacterial co-infections was conducted. This represents a limitation of the study. Future work will incorporate additional laboratory diagnostics, as the possibility of concurrent infections cannot be excluded.

Despite these limitations, the results suggest that the developed vaccine provides effective protection against BoHV-1 and BVDV.

## 5 Conclusion

Despite the limited number of animals used in this study, valuable data on the safety and immunogenicity of the developed vaccine were obtained. The dynamics of Abs allow us to make preliminary conclusions about the duration of post-vaccination immunity, which appears to last for at least 6 months. However, due to the small sample size, subsequent confirmation on an expanded sample is needed to validate these findings and develop statistically robust conclusions. In future work, we plan to evaluate the effectiveness of the vaccine under field conditions.

## Data Availability

The original contributions presented in the study are included in the article/supplementary material, further inquiries can be directed to the corresponding authors.
